# Mechanisms Regulating Insulin Response to Intragastric Glucose in Lean and Non-Diabetic Obese Subjects: A Randomized, Double-Blind, Parallel-Group Trial

**DOI:** 10.1371/journal.pone.0150803

**Published:** 2016-03-04

**Authors:** Anne Christin Meyer-Gerspach, Lucian Cajacob, Daniele Riva, Raphael Herzog, Juergen Drewe, Christoph Beglinger, Bettina K. Wölnerhanssen

**Affiliations:** Department of Biomedicine, Division of Gastroenterology, University Hospital Basel, Basel, Switzerland; Daping Hospital, Third Military Medical University, CHINA

## Abstract

**Background/Objectives:**

The changes in blood glucose concentrations that result from an oral glucose challenge are dependent on the rate of gastric emptying, the rate of glucose absorption and the rate of insulin-driven metabolism that include the incretins, glucose-dependent insulinotropic peptide (GIP) and glucagon-like peptide-1 (GLP-1). The rate of insulin-driven metabolism is clearly altered in obese subjects, but it is controversial which of these factors is predominant. We aimed to quantify gastric emptying, plasma insulin, C-peptide, glucagon and glucose responses, as well as incretin hormone secretions in obese subjects and healthy controls during increasing glucose loads.

**Subjects/Methods:**

The study was conducted as a randomized, double-blind, parallel-group trial in a hospital research unit. A total of 12 normal weight (6 men and 6 women) and 12 non-diabetic obese (BMI > 30, 6 men and 6 women) participants took part in the study. Subjects received intragastric loads of 10 g, 25 g and 75 g glucose dissolved in 300 ml tap water.

**Results:**

Main outcome measures were plasma GLP-1 and GIP, plasma glucagon, glucose, insulin, C-peptide and gastric emptying. The primary findings are: i) insulin resistance (*P* < 0.001) and hyperinsulinemia (*P* < 0.001); ii) decreased insulin disposal (*P* < 0.001); iii) trend for reduced GLP-1 responses at 75 g glucose; and iv) increased fasting glucagon levels (*P* < 0.001) in obese subjects.

**Conclusions:**

It seems that, rather than changes in incretin secretion, fasting hyperglucagonemia and consequent hyperglycemia play a role in reduced disposal of insulin, contributing to hyperinsulinemia and insulin resistance.

**Trial Registration:**

ClinicalTrials.gov NCT01875575

## Introduction

In the lean, the insulin response to an oral glucose challenge or to meal ingestion is largely influenced by the rate of gastric emptying and by the concomitant secretion of the incretin hormones, glucagon-like peptide-1 (GLP-1) and glucose-dependent insulinotropic peptide (GIP) [1–4]. Gastric emptying accounts roughly for one-third of the variance in the glycemic response, as determined with a standard 75 g glucose tolerance test in healthy controls [2]. In the small intestine, the incretin effect was much greater in response to an intraduodenal infusion of glucose at 4 kcal/min compared to 2 kcal/min indicating that the rate of small intestinal glucose exposure is another determinant of the secretion of GLP-1 and GIP [5]. In response to oral glucose, the incretin hormones increase with the size of the glucose load, resulting in similar glucose excursions, independent of the glucose load [1,6]. This regulatory adaption was explained as dose-dependent responses by both GLP-1 and GIP [1,6]. According to Nauck and coworkers [1], the incretin effect accounts for up to 70% of the insulin response to a standard 75 g oral glucose tolerance test.

In obesity, insulin resistance and hyperinsulinemia are early metabolic changes [7,8]. Both in the obese and in patients with type 2 diabetes mellitus (T2DM), the incretin effect is impaired [9]. Experimental evidence suggests that this effect is not explained by attenuated secretion of GLP-1 or GIP [9], but, more probably, is a consequence of a β-cell-sensing defect to glucose and/or GLP-1 and GIP. Contradictory results have been reported for gastric emptying showing accelerated [10–12], normal [13–15] or even delayed gastric emptying rates [16–18] in the obese.

For severe obesity which is resistant to conventional therapy, bariatric surgery is currently the only effective treatment. After Roux-en-Y gastric bypass (RYGB) surgery, glucose tolerance is markedly improved, accompanied by accelerated emptying from the gastric pouch to the distal small intestine; the distal gut is therefore exposed to greater quantities than normal of ingested nutrients [19] with exaggerated incretin hormones responses [20]. In order to better understand the different phases in the pathophysiology of improved glucose metabolism, it would be useful to perform glucose tolerance studies post-RYGB surgery. However, these patients do not tolerate high glucose loads as severe adverse events (dumping syndrome, reactive hypoglycemia) can occur.

Primary outcome of this present study was to characterize the various parameters regulating glycemia and quantify insulin, C-peptide, glucagon and glucose responses, as well as incretin hormone secretions in obese subjects without T2DM and in healthy controls during increasing glucose loads ranging from 10 g to 75 g of glucose. Secondary outcome was to investigate gastric emptying rates of the different glucose loads. In addition, the study should provide experimental evidence for the use of low glucose loads applied for research purposes in patients undergoing bariatric surgery.

## Materials/Subjects and Methods

### Subjects

A total of 12 normal weight (mean BMI: 22.0 ± 0.4 kg/m^2^, range 19.0–24.9 kg/m^2^) volunteers (6 men and 6 women; mean age: 24.3 ± 0.6 years, range 20–32 years) and 12 non-diabetic obese (mean BMI: 38.8 ± 0.9 kg/m^2^, range 30.5–48.4 kg/m^2^) participants (6 men and 6 women; mean age: 29.4.8 ± 1.8 years, range 19–48 years) took part in the study; all were healthy. Samples size of this study was chosen on the basis of practical considerations rather than statistical estimation. However, according to our experience, a sample size of 12 subjects in each group will most likely allow to detect large differences in parameters (> 50%) between the treatments groups.

### Overall study design

The study was conducted as a randomized (balanced), double-blind, parallel-group trial, and in accordance with the Declaration of Helsinki and was carried out at the Phase one Research Unit of the University Hospital of Basel. The protocol was submitted and approved by the Local Research and Ethics Committee in Basel (Ethikkommission Nordwest- und Zentralschweiz (EKNZ): 298/12; approval date: 19. December 2012). Subjects recruitment (by word of mouth) and follow-up were over a period of three months (01. Februar 2013–30. April 2013). Each subject gave written informed consent for the study. The study is registered at ClinicalTrials.gov (NCT01875575); the registration was after enrolment of participants started. The reason for this is that the study protocol is part of a large obesity and diabetes cohort study and to enter this data would have meant to share confidential, sensible information about our research plans. The authors confirm that all ongoing related trials for this intervention are registered. Exclusions included smoking, substance abuse, regular intake of medications, psychiatric or medical illness (especially diabetes) and any abnormalities detected on physical examination or laboratory screening. None of the subjects had a history of gastrointestinal disorders, food allergies or dietary restrictions. Anthropometric measurements, including weight, height, BMI, as well as heart rate and blood pressure, were recorded for all participants. Subjects were instructed to abstain from alcohol, caffeine, black- and green- tee, coke, chocolate and strenuous exercise for 24 hours before each treatment and, furthermore, to abstain from sprouts, broccoli and grapefruit for the entire study duration. After fasting overnight for at least 10 hours, participants were admitted to the Phase 1 Research Unit of the University Hospital of Basel at 0800 h. An antecubital catheter was inserted into a forearm vein for blood collection.

Subjects swallowed a radiopaque polyvinyl feeding tube (external diameter 8 French). The gastric tube was placed through an anesthetized nostril; its intragastric (ig) position was confirmed by rapid injection of 10 mL of air and auscultation of the upper abdomen. Volunteers were seated in a comfortable chair during the session. Except for the ig infusions, the test trials were identical in design. The infusions were different concentrations of glucose (10 g, 25 g or 75 g) dissolved in 300 ml tap water, with 50 mg ^13^C-sodium acetate (for determining gastric emptying). The placebo treatment was 300 ml tap water. On 4 separate occasions, at least 3 days apart, the subjects received 1 of the test solutions in random order. The solutions were freshly prepared and were at room temperature when administered. The person preparing the solution and the person administering the treatment were not identical, so that deliverer and recipient were blinded.

### Experimental procedure

After taking two fasting blood samples (t = -10 and -1 min) and a fasting breath sample (t = -1 min), subjects received the test solution via the feeding tube within 2 minutes (t = 0–2 min). After administration, the feeding tube was directly removed.

At regular time intervals (15, 30, 45, 60, 90, 120, and 180 min) after instillation of the test solution, blood samples were collected on ice into tubes containing EDTA (6 μmol/l), a protease inhibitor cocktail (Complete^®^, EDTA-free, 1 tablet/50 ml blood; Roche, Mannheim, Germany) and a dipeptidylpeptidase IV inhibitor (10 μl/ml; Millipore Corporation, St. Charles, Missouri, USA). Tubes were centrifuged at 4°C at 3000 rpm for 10 min and plasma samples were processed into different aliquots. The total blood volume taken during one test day was 100 ml. All samples were stored at -70°C until analysis of plasma active GLP-1, total GIP, PYY, glucagon, insulin, C-peptide and glucose.

For determining gastric emptying rates, end-expiratory breath samples were taken at fixed time intervals (15, 30, 45, 60, 75, 90, 105, 120, 150, 180, 210, and 240 min) after instillation of the test solution.

The subject’s vital signs (blood pressure, heart rate) were measured before and after each study intervention.

### Assessment of gastric emptying

The gastric emptying rate was determined using a ^13^C-sodium acetate breath test, an accurate, non-invasive method for measuring gastric emptying, without radiation exposure, and a reliable alternative to scintigraphy, the current “gold standard” [21]. Test solutions were labeled with 50 mg of ^13^C-sodium acetate, an isotope absorbed readily in the proximal small intestine, next transported to the liver where it is metabolized to ^13^CO_2_, which is then exhaled rapidly [21]. At fixed time intervals, end-expiratory breath samples were taken into a 100 ml foil bag. The ^13^C-exhalation was determined by non-dispersive infrared spectroscopy using an isotope ratio mass spectrophotometer (IRIS^®^; Wagner Analysen Technik, Bremen, Germany), and expressed as the relative difference (δ ‰) from the universal reference standard (carbon from Pee Dee Belemnite limestone). ^13^C-enrichment was defined as the difference between pre-prandial ^13^C-exhalation and post-prandial ^13^C-exhalation at defined time points, δ over basal (DOB, ‰). Delta values were converted into atom percent excess and then into percent of administered dose of ^13^C excreted per hour (%dose/h (%)). In this last conversion, the CO2 production of the subjects was used, which is assumed to be 300 mmol/h multiplied by the body surface area. The body surface area was calculated by the weight height formula of Haycock *et al*. [22].

### Materials

Glucose was purchased from Hänseler (Herisau, Switzerland) and ^13^C-sodium acetate was purchased at ReseaChem (Burgdorf, Switzerland).

### Laboratory analysis

#### Active GLP-1, total GIP and PYY

Active GLP-1, total GIP and PYY were measured by an Immunological Multi-Parameter Chip Technology (Roche Diagnostics GmbH, Penzberg, Germany). The intra- and inter-assay coefficients of variation are below 9.5% and 10.0%, respectively.

#### Insulin, C-peptide and glucagon

Insulin, C-peptide and glucagon were measured with commercially-available ELISA kits. The ELISA kits for insulin and C-peptide were purchased at Abnova, Taipei City, Taiwan; the ELISA kit for glucagon at Cusabio, Wuhan, China. The intra- and inter-assay coefficients of variation are below 8.1% and 8.5% (for insulin), below 6.7% and 9.9% (for C-peptide) and below 8.0% and 10.0%, respectively (for glucagon).

#### Plasma glucose

Plasma glucose concentration was measured by a glucose oxidase method (Rothen Medizinische Laboratorien AG, Basel, Switzerland). The intra- and inter-assay coefficient of variation is below 2.9% and 3.9%, respectively. After the 75 g glucose load, each subject was classified according to WHO criteria, as having impaired glucose tolerance (2 h blood glucose <11.1 mmol/l, but >7.8 mmol/l) or diabetes (fasting blood glucose ≥7.0 mmol/l and/or 2 h blood glucose ≥11.1 mmol/l) [23]. The 60 min blood glucose level after the 75 g glucose load was used as predictor of the development of type 2 diabetes (with a cut-off of 8.6 mmol/l as risk factor) [24,25].

### Calculation of insulin/C-peptide clearance

Differences in clearance of insulin and C-peptide were assessed for normal weight and obese subjects. Under the assumption that the stimulated dose of pro-insulin leads to the same molar amounts of secreted insulin and C-peptide (Dose = Dose_insulin_ = Dose_C-peptide_), the incremental iAUC (= AUC above baseline) and clearance (Cl) are related as

$$\begin{array}{c} {\text{Dose }=\text{ Cl}}_{\text{insulin}}{*\text{ iAUC}}_{\text{insulin}}{\text{ and Dose }=\text{ Cl}}_{\text{C-peptide}}{*\text{ iAUC}}_{\text{C-peptide}}\\ \text{hence}\\ {\text{Cl}}_{\text{insulin}}{*\text{ iAUC}}_{\text{insulin}}{=\text{ Cl}}_{\text{C-peptide}}{*\text{ iAUC}}_{\text{C-peptide}}\\ {\text{or}}_{}\\ {}_{\;}{\text{iAUC}}_{\text{C-peptide}}{\text{/ iAUC}}_{\text{insulin}}{=\text{ Cl}}_{\text{insulin}}{\text{/ Cl}}_{\text{C-peptide}}=\text{ R}\\ \text{and finally}\\ {\text{Cl}}_{\text{insulin}}{=\text{ R }*\text{ Cl}}_{\text{C-peptide}} \end{array}$$

where R denotes the ratio of insulin over C-peptide clearance.

### Statistical analysis

Descriptive statistics were used for demographic variables, such as age, weight, height, and BMI. Hormone and glucose profiles were analyzed by calculating the area under the concentration-time curve (AUC, 0–180 min, using the linear trapezoidal rule) from baseline values and maximal plasma concentrations (C_max_). Insulin resistance was quantified using the HOMA-IR index (Table 1) by the formula: (fasting glucose*fasting insulin)/22.5. Gastric emptying rates were analyzed by calculating the AUC (0–240 min). The parameters were tested for normality by the Shapiro-Wilk test method. Dose-response with regard to increasing glucose loads was analyzed by repeated measures ANOVA and simple contrasts with Bonferroni correction. In case of non-normally distributed data, analysis was performed on log-transformed data; Tmax was tested by Friedman’s test and multiple Wilcoxon signed rank tests between the groups, when necessary.

**Table 1:** Effect of different glucose loads on plasma glucose, insulin and glucagon levels.

Hormones and HOMA-IR			Lean controls	Obese	Significance
**Plasma glucose**	**Fasting glucose (mmol/l)**		4.9 ± 0.1	5.2 ± 0.1	*P = 0*.*005*
	**Cmax (mmol/l)**	10 g glucose	6.6 ± 0.1	6.6 ± 0.2	*NS*, *P = 0*.*948*
		25 g glucose	7.4 ± 0.3	7.7 ± 0.2	*NS*, *P = 0*.*340*
		75 g glucose	7.8 ± 0.2	9.0 ± 0.3	*P = 0*.*006*
	**iAUC (0–180 min, mmol x min/l)**	10 g glucose	21.9 ± 18.4	-3.3 ± 7.7	*NS*, *P = 0*.*224*
		25 g glucose	75.0 ± 26.2	59.8 ± 13.2	*NS*, *P = 0*.*610*
		75 g glucose	108.3 ± 36.9	251.6 ± 31.4	*P = 0*.*007*
**Plasma insulin**	**Fasting insulin (μU/ml)**		4.3 ± 0.5	15.2 ± 1.4	*P < 0*.*001*
	**Cmax (μU/ml)**	10 g glucose	15.6 ± 2.5	45.6 ± 4.0	*P < 0*.*001*
		25 g glucose	24.5 ± 3.3	82.3 ± 9.9	*P < 0*.*001*
		75 g glucose	39.9 ± 5.9	101.5 ± 10.6	*P < 0*.*001*
	**iAUC (0–180 min, μU x min/ml)**	10 g glucose	99.8 ± 64.3	280.7 ± 176.2	*NS*, *P = 0*.*351*
		25 g glucose	656.5 ± 128.4	2959.7 ± 322.3	*P < 0*.*001*
		75 g glucose	2791.8 ± 380.0	7747.2 ± 1246.3	*P = 0*.*002*
**HOMA-IR**			1.0 ± 0.1	3.5 ± 0.3	*P < 0*.*001*
**Plasma glucagon**	**Fasting glucagon (pg/ml)**		32.6 ± 3.4	66.7 ± 4.2	*P < 0*.*001*

iAUC, incremental area under the concentration-time curve; C_max_, maximum plasma concentration. Data are expressed as mean ± SEM. n = 12 lean controls and 12 obese subjects. *P* ≤ 0.05, statistically significant difference vs. lean controls; NS, not statistically significant vs. lean controls.

Student’s unpaired t-test or Mann-Whitney test was used to test for significant differences between lean subjects and obese participants at the different (10g, 25g and 75 g) glucose strengths, as appropriate. Dose-response analysis was done by linear or non-linear regression analysis. For the latter, a Sigmoid Emax model was applied:

$$E=\;E_{0}+\frac{E_{max}\times Dose^{N}}{ED_{50}{}^{N}+Dose^{N}},$$

where E denotes the respective hormonal effect; E_0_, the baseline effect; Emax, the maximum hormonal effect possible; Dose, the glucose dose; ED_50_, the glucose dose that produces a half-maximal hormonal effect; and N, the sigmoidicity coefficient. Regression analysis was performed by OriginPro software (OriginLab Northampton MA, Version 8.0).

All statistical analysis was done using the statistical software package, SPSS for Windows, Version 22.0 (SPSS Inc., Chicago, USA). Values were reported as mean ± SEM (standard error of the mean). Differences were considered to be significant when *P* < 0.05.

## Results

**Fig 1:**
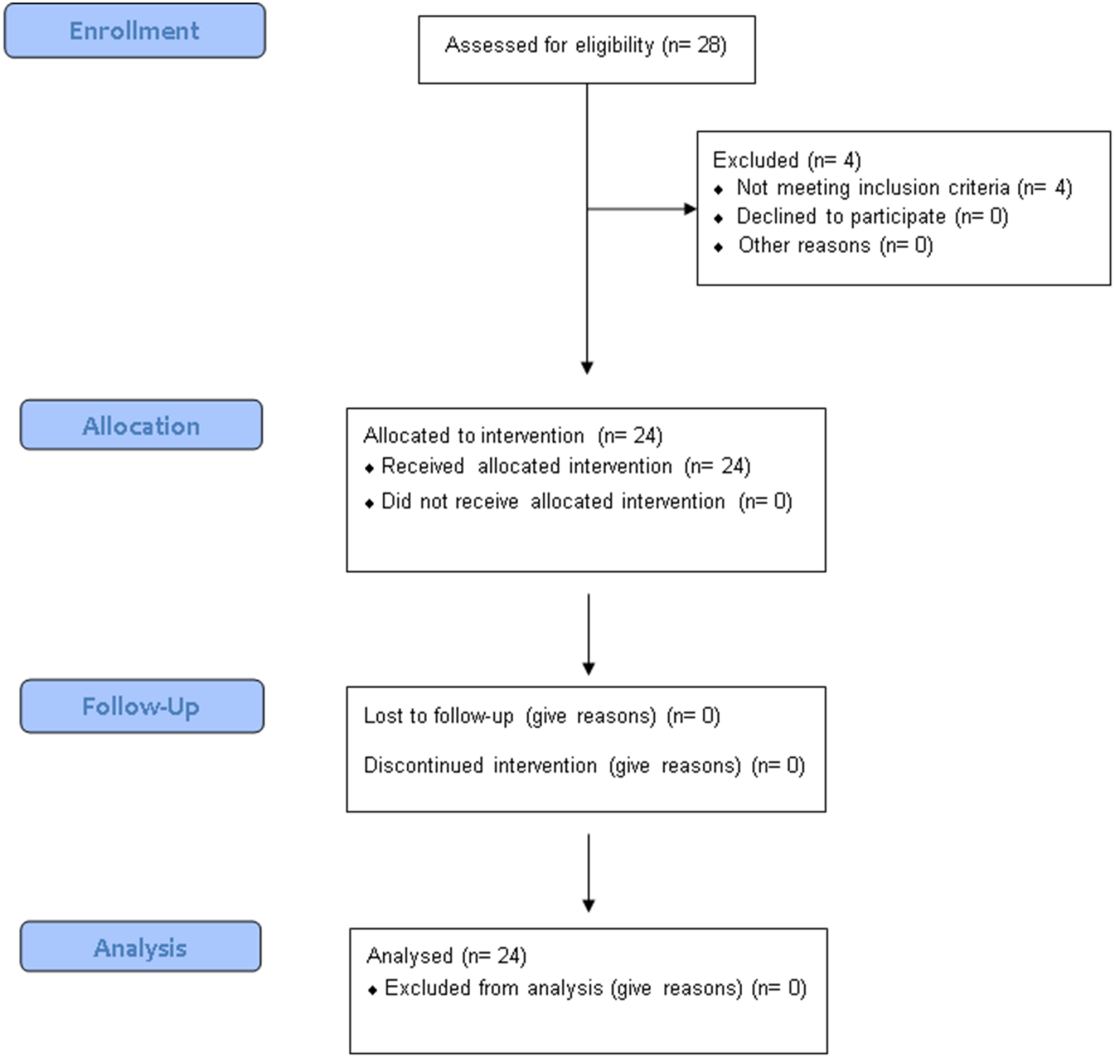
Flowchart. Adapted CONSORT flowchart for clinical trials.

All subjects tolerated the study well and there were no adverse events. None of the subjects had diabetes, one subject had impaired glucose tolerance and four had a risk factor for diabetes (according to the 60 min blood glucose levels after 75 g glucose). There was no drop-out; complete data from 24 subjects (12 lean and 12 obese) were obtained for analysis **(**Fig 1**)**.

### Fasting and postprandial glucose levels

**Fig 2:**
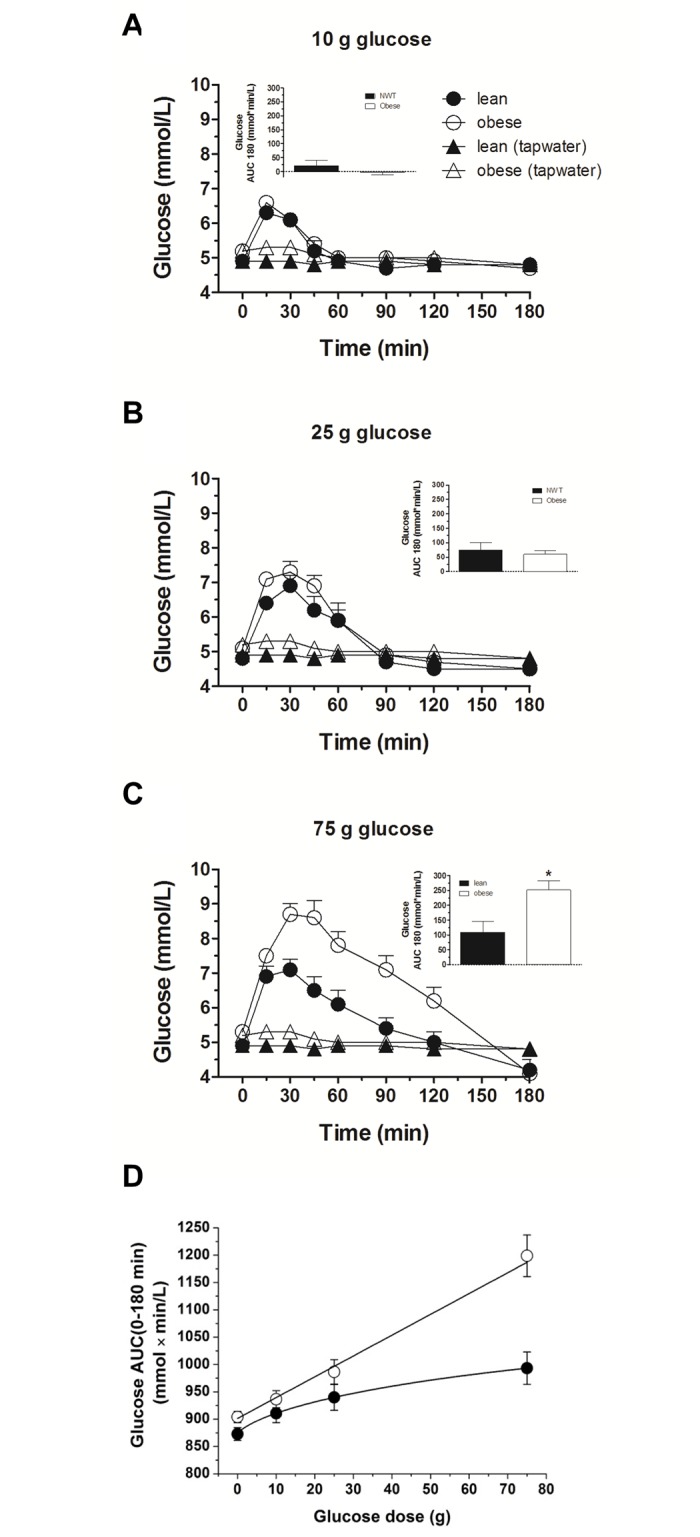
Plasma glucose. Plasma glucose concentrations in response to ig loads of 10 g (A), 25 g (B) and 75 g (C) of glucose as well as dose-responses to ig glucose loads (D) in lean and obese subjects. AUC, area under the concentration-time curve. Data are expressed as mean ± SEM. *, *P ≤* 0.05, statistically significant difference vs. lean participants.

In obese subjects, fasting plasma glucose levels were significantly increased (*P* = 0.005, Table 1) compared to lean control subjects. As illustrated in Fig 2A–2C, increasing loads of glucose induced a protracted decrease in plasma glucose concentrations, both in normal weight and in obese subjects. In the control group, peak plasma glucose concentrations were similar in response to the increasing glucose loads, whereas obese subjects showed increased peak plasma glucose levels with increasing glucose loads (Fig 2A–2C, Table 1). Blood glucose was less than baseline at t = 120 and 180 min (after 25 g glucose load; *p* = 0.003 for both time points), and at t = 180 min (after 75 g glucose load; *p* = 0.029) in the control group. In the obese group, blood glucose was less than baseline at t = 60, 90, 120 and 180 min (after 10 g glucose load; *p* < 0.01 for all), at t = 120 and 180 min (after 25 g glucose load; *p* < 0.001 for both), and at t = 180 min (after 75 g glucose load; *p* < 0.001). We infer from these observations that obese subjects can still control blood glucose levels at low glucose challenges; alternatively, we cannot exclude that the difference at these time points is caused by the higher baseline glucose concentrations. This is supported by the observation that at the lower two glucose loads (10 and 25 g), integrated plasma glucose levels were not different between obese subjects and lean controls (Fig 2A, 2B and 2D, Table 1). However, after 75 g glucose administration, plasma glucose levels were significantly higher in the obese group compared to the lean counterparts (iAUC 0–180 minutes: *P* < 0.007, Fig 2C and 2D, Table 1).

### Fasting and postprandial insulin and C-peptide levels

**Fig 3:**
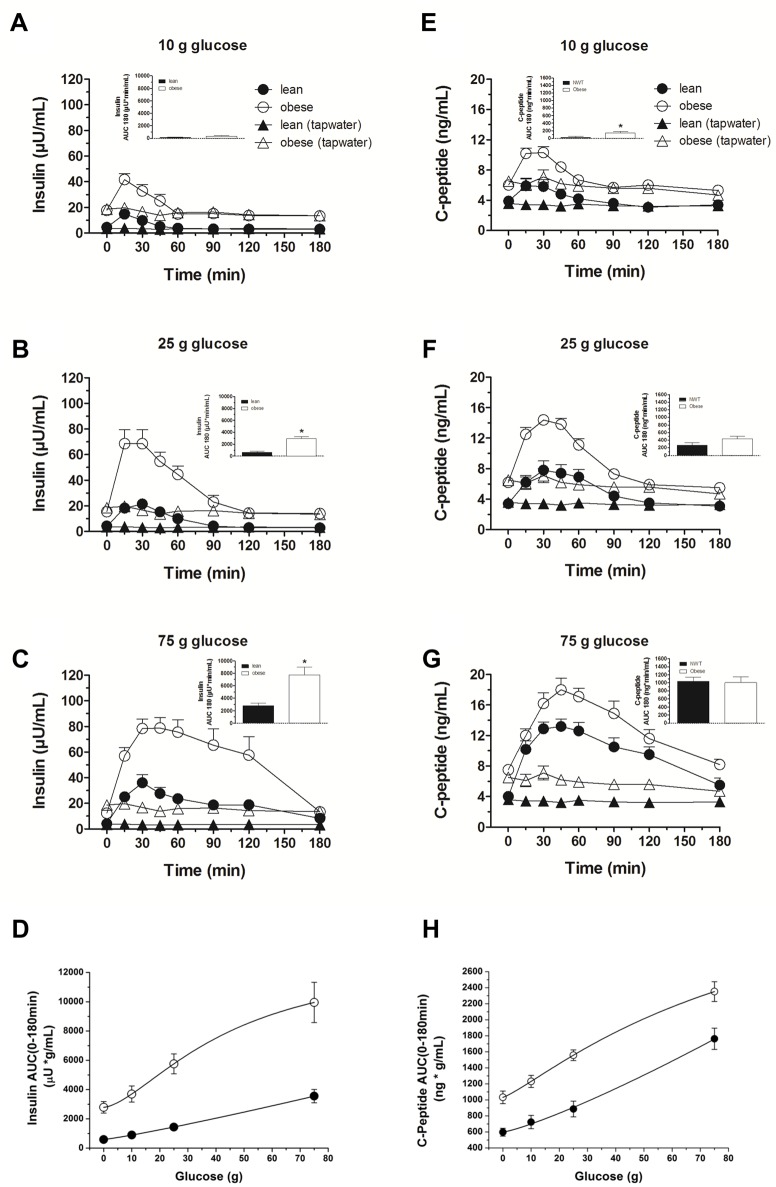
Plasma insulin and C-peptide. Plasma insulin and C-peptide concentrations in response to ig loads of 10 g (A, E), 25 g (B, F) and 75 g (C, G) of glucose as well as dose-responses to ig glucose loads (D, H) in lean and obese subjects. AUC, area under the concentration-time curve. Data are expressed as mean ± SEM. *, *P ≤* 0.05, statistically significant difference vs. lean participants.

In obese subjects, fasting insulin and C-peptide levels were significantly increased (*P* < 0.001, respectively, Table 1 (insulin)). Time courses for insulin and C-peptide levels are depicted in Fig 3A–3C and 3E–3G, respectively, and Table 1 (insulin): In both subject groups, increasing loads of glucose administration induced a dose-dependent rise in plasma insulin and C-peptide levels, but the slope of the two curves was different (Fig 3D and 3H). Plasma insulin levels were significantly higher in obese subjects after the 25 g and 75 g glucose loads (iAUC 0–180 minutes: *P* < 0.001 and *P* = 0.002, respectively) compared to the lean controls.

By calculating the ratio of incremental AUC for insulin and C-peptide, we estimated insulin clearance for both lean and obese subjects. The results document that the insulin:C-peptide molar ratio was significantly reduced in the obese group at 25 g and 75 g glucose (*P* = 0.015 and *P* < 0.001, respectively) compared to normal weight subjects (Table 2). We infer from these data that the obese have a decreased insulin disposal.

**Table 2:** Ratio (R) of insulin over C-peptide clearance in response to different glucose loads (10 g, 25g and 75 g) in lean and obese healthy subjects.

Glucose (in g)	Lean controls	Obese	Significance
**10**	21.5 ± 26.2	- 1.7 ± 22.6	NS, *P* = 0.509
**25**	18.6 ± 3.6	7.5 ± 1.3	*P* = 0.015
**75**	19.9 ± 2.4	6.6 ± 0.8	*P* < 0.001

### Fasting glucagon levels

In obese subjects, fasting glucagon levels were significantly increased (*P* < 0.001, Table 1).

### Fasting and postprandial GI peptide levels

No differences in fasting GLP-1 (lean: 13.6 ± 1.1 pg/mL; obese: 15.3 ± 1.6 pg/mL, *P = 0*.*304*), PYY (lean: 20.3 ± 1.3 pg/mL; obese: 19.6 ± 1.5 pg/mL, *P = 0*.*770*) or GIP (lean: 143.8 ± 15.5 pg/mL; obese: 127.9 ± 3.2 pg/mL, *P = 0*.*337*) levels were observed between obese subjects and the lean controls.

**Fig 4:**
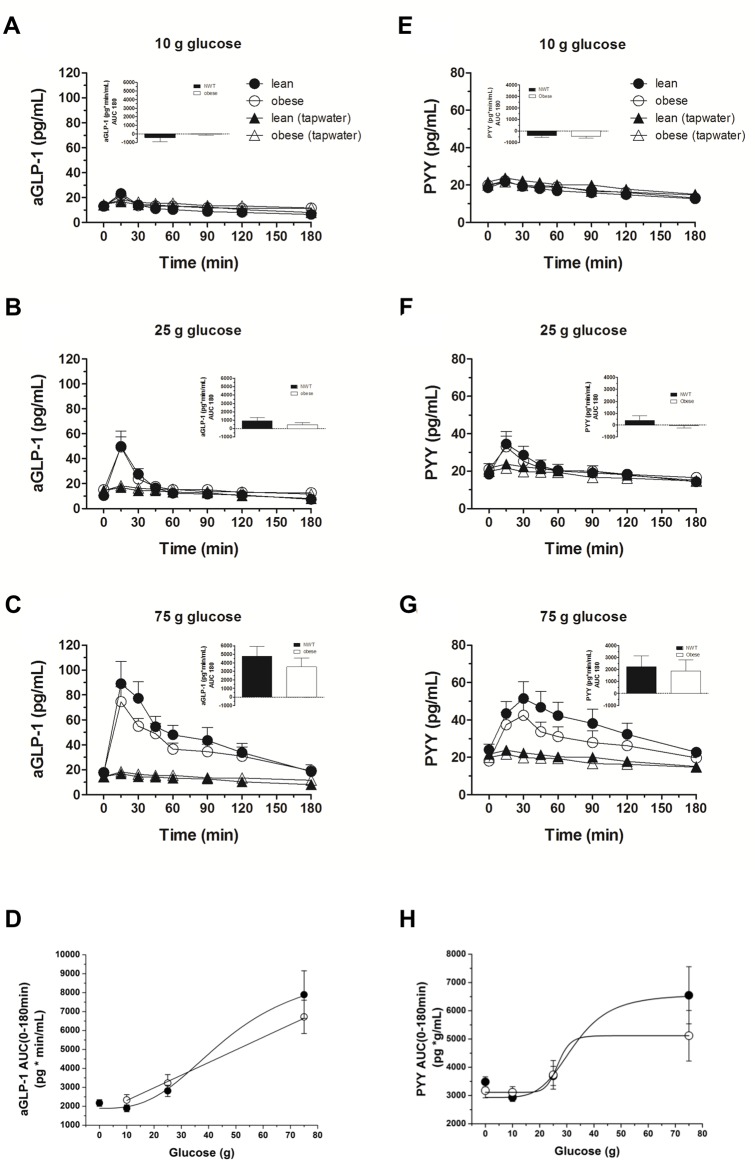
Plasma aGLP-1 and PYY. Plasma GLP-1 and PYY concentrations in response to ig loads of 10 g (A, E), 25 g (B, F) and 75 g (C, G) of glucose as well as dose-responses to ig glucose loads (D, H) in lean and obese subjects. aGLP-1, active glucagon-like peptide-1; PYY, peptide tyrosine tyrosine; AUC, area under the concentration-time curve. Data are expressed as mean ± SEM. *, *P ≤* 0.05, statistically significant difference vs. lean participants.

The time courses for GLP-1 and PYY are depicted in Fig 4: In both subject groups, glucose administration induced a dose-dependent rise in plasma GLP-1 and PYY levels (Fig 4D and 4H). The figures illustrate that the GLP-1 and PYY levels were not different between obese subjects and lean controls after 10 g and 25 g glucose (Fig 4A, 4B, 4E and 4F). There was a trend toward decreased GLP-1 and PYY secretion in obese subjects after the 75 g glucose administration, though the effects did not reach statistical significance (Fig 4C and 4G).

**Fig 5:**
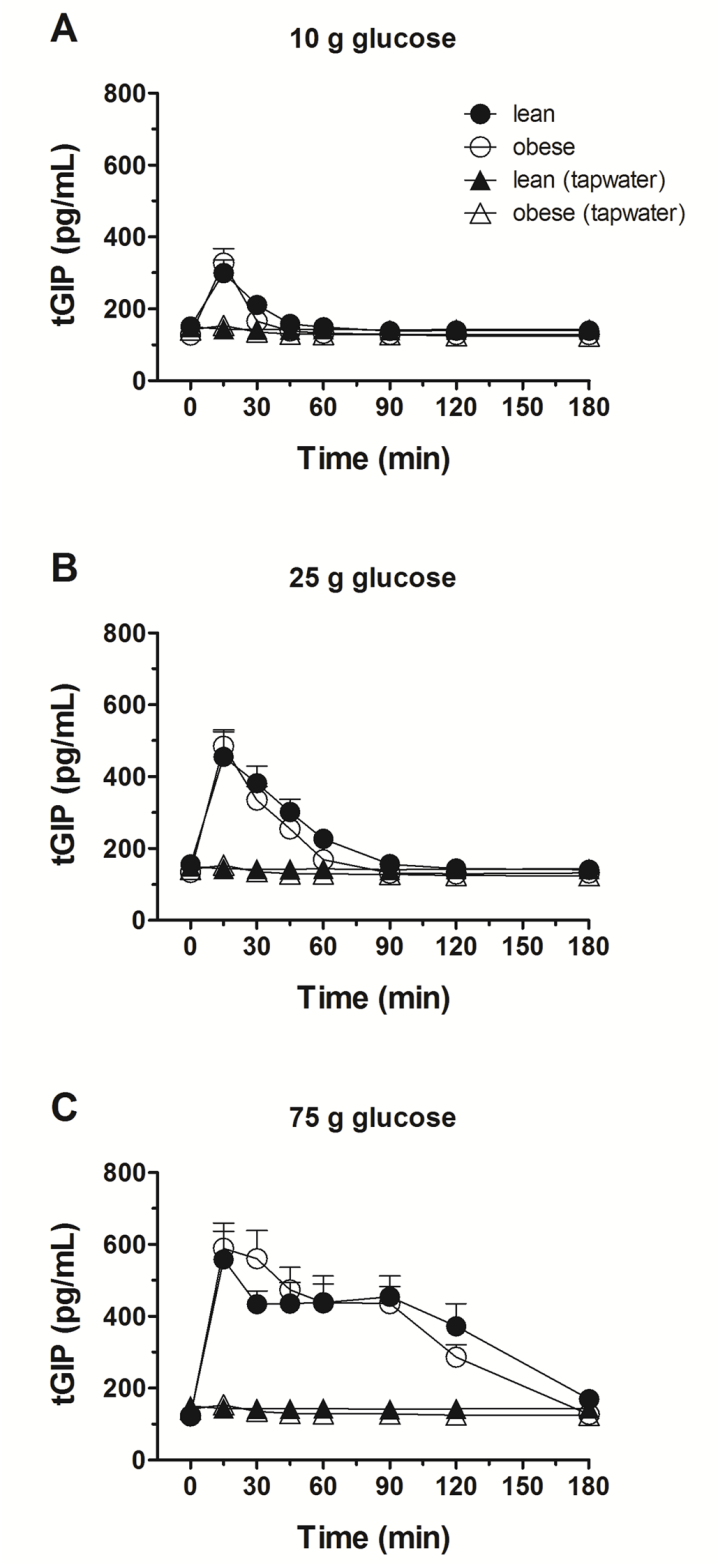
Plasma GIP. Plasma GIP concentrations in response to ig loads of 10 g (A), 25 g (B) and 75 g (C) of glucose in lean and obese subjects. tGIP, total glucose-dependent insulinotropic peptide; AUC, area under the concentration-time curve. Data are expressed as mean ± SEM. *, *P ≤* 0.05, statistically significant difference vs. lean participants.

Glucose administration induced a dose-dependent rise in plasma GIP levels in both groups, but there was no difference in the time courses of plasma GIP levels between obese subjects and lean controls after all three glucose loads (Fig 5A–5C).

### Gastric emptying rates

**Fig 6:**
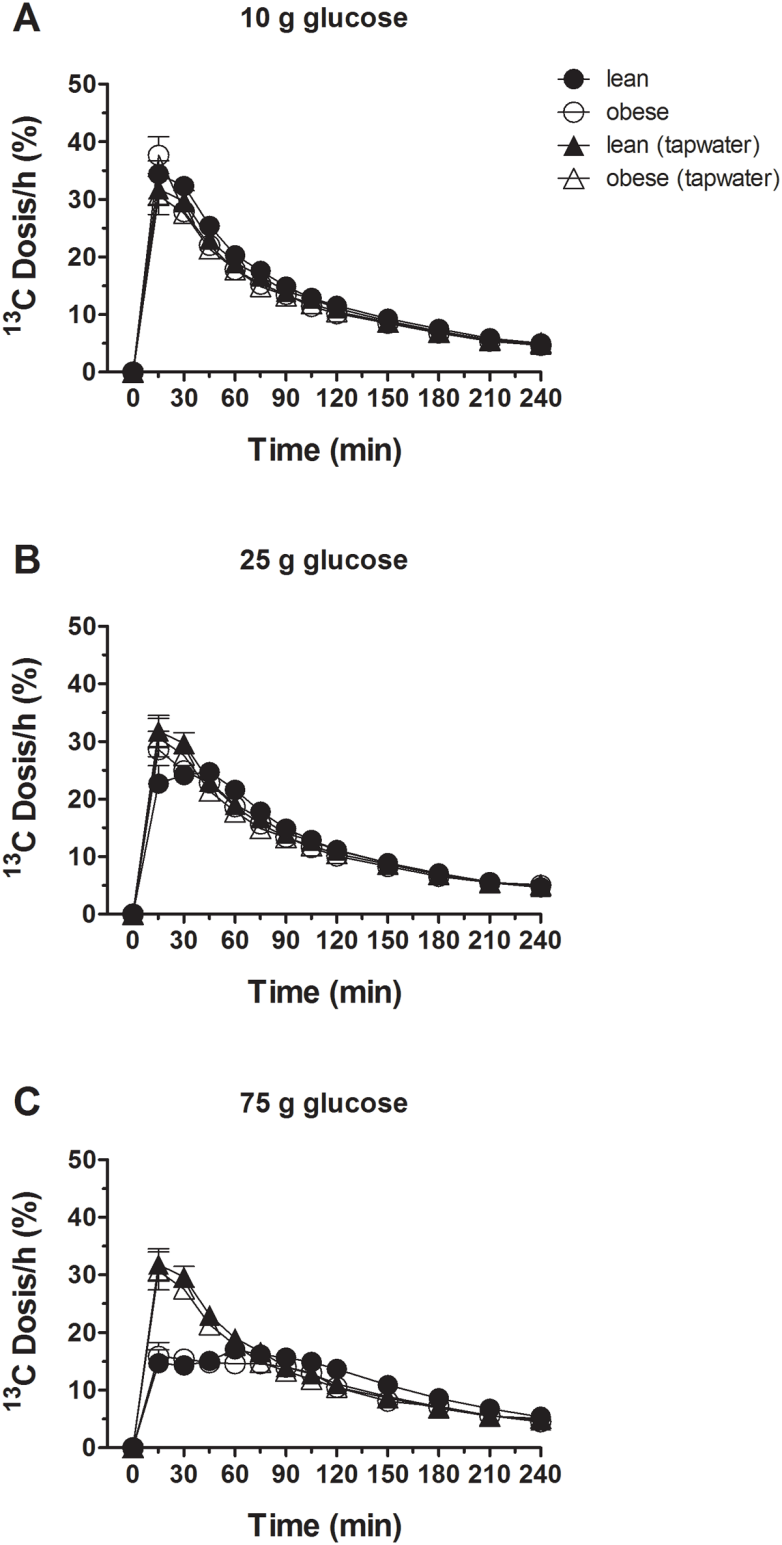
Gastric emptying rates. Gastric emptying rates in response to ig loads of 10 g (A), 25 g (B) and 75 g (C) of glucose in lean and obese subjects. Data are expressed as mean ± SEM.

Increasing amounts of glucose induced a prolonged gastric emptying time in both subject groups, but there were no differences in gastric emptying rates between obese subjects and lean controls after all three glucose loads (Fig 6A–6C).

## Discussion

The changes in blood glucose concentrations evoked by an oral glucose challenge are dependent on the rate of gastric emptying, the rate of glucose absorption and the rate of insulin-driven metabolism that include the incretin hormones, GIP and GLP-1 [2]. The rate of insulin-driven metabolism is clearly altered in obese subjects, but it is controversial which of these factors is predominant. With this background in mind, the present study was designed to comprehensively analyze the different parameters in regulating blood glucose concentrations in response to increasing glucose loads (ranging from threshold to a standard 75 g glucose), both in lean and obese subjects.

The primary findings of the present study using small (10 g) to standard (75 g) glucose loads are: i) insulin resistance and hyperinsulinemia; ii) decreased insulin disposal (*P* < 0.001); iii) trend for reduced GLP-1 responses at 75 g glucose and unchanged GIP responses; and iv) increased fasting glucagon levels in obese, non-diabetic subjects compared to lean controls. Details on the new findings are discussed below.

### Gastric emptying

There was a load-dependent slowing of gastric emptying, both in lean and obese subjects. The gastric emptying rates were similar in both groups. Gastric emptying rates have been reported to be comparable, faster, or slower in obese subjects compared to lean persons [13]. The reasons for the discrepant results can be attributed to a variety of factors including different methods for quantifying gastric emptying, differences in nutrient composition and meal characteristics (liquid vs solid) and metabolic state and limited number of subjects studied under various conditions. Here we used the ^13^C-sodium acetate breath test and liquid glucose drinks to assess gastric emptying. Gastric emptying of glucose drinks is primarily dependent on neural and humoral feedback loops triggered by the interaction of glucose with sensing mechanisms in the small intestine. In this study, the humoral responses (GIP, GLP-1, PYY) were similar in lean and obese subjects reflecting the gastric emptying patterns in both groups. Given the limited number of subjects that were evaluated (n = 12 in each group), we cannot exclude the possibility of differences in gastric emptying rates between lean and obese persons.

### Glucose, insulin and C-peptide

The study also shows that fasting insulin and glucose concentrations are significantly increased in obese subjects, results are in line with previous findings [26]; the HOMA-Index (indicator for insulin resistance) was significantly (*P* < 0.001) elevated. There was a rise in blood glucose in response to all three glucose loads, with no difference in blood glucose concentrations between obese and lean controls after the 10 and 25 g glucose loads; in contrast, after the 75 g glucose load a significantly elevated increase in blood glucose levels was observed in obese compared to lean controls. These novel findings suggest the existence of a threshold for a glucose challenge—above this value, obese subjects are no longer able to adapt to increasing plasma glucose levels with proportionate changes in insulin secretion. A progressive, dose-dependent rise in plasma insulin and C-peptide occurred (as assessed by AUCs and peak concentrations) in response to all three glucose loads, both in healthy and obese subjects; the increase in plasma insulin was significantly higher in the obese group (and this occurred even at low glucose loads). Together, these findings confirm that obese subjects have an impaired glucose homeostasis and exhibit prediabetic factors, including hyperinsulinemia and insulin resistance.

We used peripheral insulin:C-peptide molar ratios of incremental areas under the curve to estimate insulin disposal with the assumption that hepatic extraction of C-peptide is negligible, while the liver is a major site of insulin extraction (first-pass effect); changes in the molar ratio would therefore reflect changes in hepatic insulin extraction [27]. These assumptions have some limitations and there is no agreement in the literature as to whether fractional hepatic insulin removal is altered by administration of different concentrations of oral glucose [28]. The same authors have reported that clearance of endogenous insulin decreases progressively with increasing glucose loads. Given these limitations, we observed higher peripheral plasma insulin levels and a reduced insulin clearance in obese participants; the difference in the molar ratios between lean and obese subjects was apparent for both the 25 g and 75 g glucose load. We infer from these results that a reduced clearance of insulin is a factor that contributes to the pathogenesis of the hyperinsulinemia after an oral glucose challenge in obese subjects, thereby confirming previous reports [28,29].

### Incretins

There was a transient, modest (non-significant) rise in GLP-1 in response to 10 g of glucose; a sustained and prolonged elevation was seen with 75 g glucose, supporting previous observations that a significant increase in GLP-1 secretion requires a minimal small intestinal calorie load of glucose: Schirra *et al*. [30] have suggested that a threshold glucose delivery of ~2 kcal/min be exceeded in order for GLP-1 secretion to occur in healthy subjects; other studies support these conclusions [31–33]. We extend these observations to obese subjects. No significant difference in GLP-1 responses was noted at the lower two glucose loads between lean and obese subjects, but at 75 g of glucose an attenuated response was seen in obese subjects. We and others have previously reported that obese adolescent and adult persons have an attenuated GLP-1 response to meal ingestion compared to lean persons [34–36]. The attenuated response is not due to an impaired GLP-1 response to specific amino acids ingestion (Meyer-Gerspach *et al*.; publication in press). Here, no significant difference in GLP-1 secretion was observed between lean and obese persons—however a trend for reduced secretion in obese after 75 g glucose was shown. It may be that the sample size is not big enough to perceive small but significant difference. Another possibility is that, contrary to our expectations, the attenuated GLP-1 response to meal intake is not mediated by an impaired response to glucose and an alternative mechanism is responsible for reduced meal-stimulated GLP-1 secretion in obesity. The effect could be due to a reduced response to luminal long-chain fatty acids; long-chain fatty acids have a critical role in various digestive processes such as bile and pancreatic enzyme secretion and appetite regulation [37,38]; they also act as signalling molecules for the release of several hormones including CCK, GLP-1, PYY and neurotensin [39]. One sensor for long-chain fatty acids is the G-protein-coupled receptor GPR120 (also known as O3FAR1); the receptor has a key role in the control of energy balance [40]; dysfunction of the receptor could lead to reduced effects including attenuated hormone release such as GLP-1. This last conclusion needs to be confirmed by experimental evidence.

There was a load-dependent stimulation of GIP in both groups with no significant differences between the two groups. GIP has been proposed to be responsible for the insulin response to lower small intestinal glucose loads [41]; the time courses of GIP and insulin secretion seen in the present study are compatible with this hypothesis: Even at 10 g of glucose, an increase in GIP was observed in both the lean and obese.

Together these findings indicate that the observed high insulin levels in the obese group cannot be accounted for by differential secretion of GLP-1 and GIP, as the two incretin hormones were secreted in a similar fashion in both groups.

### Glucagon

As mentioned before, fasting glucagon levels were significantly increased in obese subjects. Knop *et al*. suggested that fasting hyperglucagonemia develops before glucose intolerance and the development of T2DM [9], and that high fasting glucagon levels exacerbate postprandial hyperglycemia and, as a consequence, could induce an increase in plasma insulin and subsequent hyperinsulinemia as well as insulin resistance [42]. These assumptions are in line with our results obtained in obese adolescents where fasting glucagon levels were positively associated with insulin resistance [36]. The reasons for fasting hyperglucagonemia might be a reduced glucagon-suppressing action of: i) GLP-1 (concept of incretin resistance) [43,44] and/or ii) insulin (concept of insulin resistance) in the α-cell [45].

Limitations of this study are: i) the measurement of gastric emptying by the ^13^C-sodium acetate breath test. The validity of the test procedure has not achieved universal acceptance; the main reason for this reluctance is the fact that the ^13^C-breath test is an indirect measure of gastric emptying. Several publications have, however, documented that the ^13^C-breath test is a reliable alternative to scintigraphy: [46–49]. The accuracy of the breath test was recently confirmed by magnetic resonance imaging: the authors of the study observed that ^13^CO_2_ emptying with ^13^C acetate as a label reflects the dynamics of meal emptying from the stomach [48]. Large-scale studies (with more than 1200 patients) support the assumption that the ^13^C-breath test as used in this study may serve as a reliable marker of gastric emptying velocity [49]. ii) In the current study habitual energy intake was not evaluated. Previous studies have shown that dietary patterns may influence gastric emptying of glucose [50,51]. The discrepancies concerning the impact of obesity on gastric emptying, with studies showing also normal [13–15] or accelerated [10–12] gastric emptying rates, may potentially reflect differences in habitual energy intake which in most studies has not been quantified. iii) Glucose kinetics (such as absorption, endogenous production) were not assessed in the current study. Nguyen *et al*. [52] observed increased sodium dependent glucose co-transporter 1 (SGLT1) expression, accelerated glucose absorption, and positive associations between SGLT1 and glucose absorption in morbidly obese persons. Further evaluation of a potential dysregulation of intestinal glucose transporter expression and absorption in obesity is needed.

The present study has several strengths: Gastric emptying, together with the assessment of incretin concentrations were measured in response to different glucose loads in two distinct groups, representing a broad spectrum of insulin sensitivity and glucose tolerance.

Based on these results, we conclude that obese, non-diabetic subjects can be metabolically characterized by elevated fasting glucagon levels, as well as hyperinsulinemia and insulin resistance. It seems that, rather than changes in GI peptide secretion, fasting hyperglucagonemia and consequent hyperglycemia play a role in reduced disposal of insulin, contributing to hyperinsulinemia and insulin resistance. The study established that increasing loads of glucose induce variations in the delivery of glucose to the duodenum with varying effects on blood glucose, insulin, C-peptide and incretin responses, both in lean and in obese subjects without T2DM. These observations have implications for a better understanding of the regulation of postprandial glycemia. We could show that even low glucose doses can be used for research purposes. The results are especially useful for designing experiments in patients after RYGB bariatric surgery, where only small glucose loads can be administered lest severe adverse events arise.

## Supporting Information

S1 CONSORT Checklist CONSORT Checklist. (DOC)

S1 Protocol Trial Protocol. (DOCX)
