# Supplementary material for: Mechanisms Regulating Insulin Response to Intragastric Glucose in Lean and Non-Diabetic Obese Subjects: A Randomized, Double-Blind, Parallel-Group Trial
Source: PLoS One. 2016 Mar 4;11(3):e0150803. doi: 10.1371/journal.pone.0150803 (PMC4778796; doi:10.1371/journal.pone.0150803)
Supplement: S1 Protocol — (DOCX) [file pone.0150803.s002.docx]

**Influence of different concentrations of glucose on the release of gastrointestinal satiation peptides in healthy normal weight and obese humans**

Investigators: Dr. med. Bettina Woelnerhanssen

Dr. Anne Christin Meyer-Gerspach

Sponsor: Klinik für Gastroenterologie, Christoph Beglinger

**1. Introduction**

After gastric bypass many patients suffer from early and/or late dumping syndrome as a reaction to carbohydrate rich meals. Gastric emptying after bypass is accelerated and nutrition enters the intestine faster, which leads to osmotically driven fluid shifts from the blood to the lumen. Late dumping occurs 1–3 h after eating, and is caused by hyperinsulinemia and is therefore characterized by symptoms of hypoglycemia like weakness, sweating, and dizziness. In most studies examining satiation hormones after oral glucose stimulation in non-operated patients, glucose loads of 50-75 g are used. For a measurable GLP-1 rise a threshold of 2 kcal/min into the intestine is needed. After gastric bypass gastric emptying is accelerated and less glucose is necessary to reach this threshold. In an ongoing pilot project in patients after gastric bypass, we examine whether glucose loads of 10 g and 25 g induce a reliable secretion of satiation peptides without dumping symptoms. The effect of these low concentrations of glucose on peptide release as well as gastric emptying of these glucose loads in normal weight and obese (non-operated) subjects is not yet know.

**2. Objective**

The aim of this study is to examine the effect of different concentrations of glucose on the release of gastrointestinal satiation peptides (PYY, GLP-1, GIP and ghrelin), as well as on plasma insulin, c-peptide, glucose and glucagon levels in healthy normal weight and obese humans. In addition we examine the gastric emptying rates of these different glucose loads.

**3. Methods**

**3.1 Study subjects**

12 healthy normal weight and 12 healthy obese subjects will be recruited. The screening procedure will include the following assessments: a medical interview and a full physical examination. Anthropometric measurements including weight, height, BMI, as well as heart rate and blood pressure will be recorded for all participants.

The following inclusion criteria are applied:

- Healthy normal weight subjects with a body-mass index of 19.0-24.5 or healthy obese subjects with a body-mass index of > 30
- Normal eating habits
- Age 18-40 years
- Stable body weight for at least three months

Exclusion criteria:

- Smoking
- Substance abuse
- Regular intake of medications (except for oral contraceptives)
- Medical or psychiatric illness
- Any abnormality detected during the screening procedure
- history of gastrointestinal disorders
- food allergies

The protocol will be submitted and approved by the Human Ethical Research Committee of the University of Basel. All subjects will have to give written informed consent.

**3.2 Study protocol**

**Overall design**

The study will be performed as a randomized, placebo-controlled, four-way crossover study, with each subject studied on four test days at least three days apart. The test trials will be identical in design except for the administered test solution (Figure 1).

**Experimental procedure**

Subjects will be admitted to the Phase 1 Research Unit of the University Basel in the morning after fasting overnight for at least 10 hours. An antecubital vein catheter will be inserted for blood drawing and a gastric tube will be swallowed. Fasting and postprandial blood samples will be taken from all participants. After taking the fasting samples (-10, -1 min), each patient will receive 300 mL of a test solution (tap water) that contains either 10 g glucose, 25 g glucose, 75 g glucose or nothing (placebo control) (Table 1). Each test solution is labeled with 50 mg ^13^C-sodium acetate for determination of gastric emptying rate. Blood will be drawn at the following time points: 15, 30, 45, 60, 90, 120, and 180 min. A 10 mL samples will be taken at each time point into tubes containing EDTA (6 µmol/l), a protease inhibitor cocktail (Complete^®^, EDTA-free, 1 tablet/50 ml blood; Roche, Mannheim, Germany) and a dipeptidylpeptidase IV inhibitor (10 μl/ml; Millipore Corporation, St. Charles, Missouri, USA). The total blood volume taken during one test day will be 100 mL. After centrifugation, plasma samples will be kept frozen at -20°C until analysis. Glucose and the following peptides will be measured: insulin, c-peptide, glucagon, PYY, GLP-1, GIP and ghrelin. For the determination of the gastric emptying rate, breath samples will be taken at -1, 15, 30, 45, 60, 75, 90, 105, 120, 150, 180, 210 and 240 min after intake of the test solution. Breath samples will be collected in foil bags.

**Figure 1 Nutritional composition of test solutions**

|  | **Amount of Sweetener (g)** | **Energy Content (kcal)** |
| --- | --- | --- |
| **tap water** | 0 | 0 |
| **glucose** | 10 | 40 |
| **glucose** | 25 | 100 |
| **glucose** | 75 | 300 |

http://www.caloriecontrol.org/sucralos.html

**3.3 Assessment of gastric emptying**

Gastric emptying rates will be assessed using the ^13^C-sodium acetate breath test. This test is an accurate, non-invasive, simple method without radiation exposure, and represents a reliable alternative to scintigraphy, the gold standard for measuring gastric emptying (1, 2). The test solutions will be labelled with 50 mg ^13^C-sodium acetate; the substrate is rapidly absorbed in the proximal small intestine, metabolized in the liver with the production of ^13^CO_2_ which is exhaled rapidly, thus, reflecting gastric emptying of nutrients (1, 2). Subjects will be asked to exhale through a mouth-piece to collect an end-expiratory breath sample into a 100 mL foil bag at certain time intervals. The ^13^CO_2_ breath content will be determined by non-dispersive infrared spectroscopy using an isotope ratio mass spectrophotometer (IRIS; Wagner Analysen Technik, Bremen, Germany). ^13^C-abundance in breath is expressed as relative difference (δ ‰) from the universal reference standard (carbon from Pee Dee Belemnite limestone). ^13^C-enrichment is defined as the difference between preprandial ^13^C-abundance in breath and ^13^C-abundance at the defined time points postprandially and is given in δ over basal (DOB, ‰). Based on these values, time to reach maximal emptying speed and areas under the curve of the responses will be calculated (2, 3).

**3.4 Laboratory Analysis**

*Active GLP-1, total GIP and PYY* will be measured by an Immunological Multi-Parameter Chip Technology (Roche Diagnostics GmbH, Penzberg, Germany). The intra- and inter-assay coefficients of variation are below 9.5% and 10.0%, respectively.

*Total ghrelin* will be measured with a commercially available kit (Millipore Corporation, Billerica, Massachusetts, USA). The intra- and inter-assay coefficients of variation are below 10.0% and 14.7%, respectively.

*Insulin, C-peptide and glucagon* will be measured with commercially-available ELISA kits. The ELISA kits for insulin and C-peptide will be purchased at Abnova, Taipei City, Taiwan; the ELISA kit for glucagon at Cusabio, Wuhan, China. The intra- and inter-assay coefficients of variation are below 8.1% and 8.5% (for insulin), below 6.7% and 9.9% (for C-peptide) and below 8.0% and 10.0%, respectively (for glucagon).

*Plasma glucose concentration* will be measured by a glucose oxidase method (Rothen Medizinische Laboratorien AG, Basel, Switzerland). The intra- and inter-assay coefficient of variation is below 2.9% and 3.9%, respectively.

**3.5 Statistical Analysis**

Descriptive statistics will be used for demographic variables such as age, weight, height and BMI. Data of plasma hormones will be evaluated by area and plasma concentration time curves (AUC) and maximum plasma concentrations (Cmax) assessed by linear regression analysis. All statistical analysis will be done using SPSS for windows software (version 22.0). Values will be reported as mean ± SEM. Differences will be considered as significant when P ≤ 0.05.

**4. Materials**

Glucosum anhydricum will be purchased at Hänseler AG, Herisau, Switzerland.

^13^C-Sodium Acetate will be purchased at ReseaChem GmbH, Burgdorf, Switzerland.

**5. References**

1. Braden B, Adams S, Duan LP, Orth KH, Maul FD, Lembcke B, et al. The [13C]acetate breath test accurately reflects gastric emptying of liquids in both liquid and semisolid test meals. Gastroenterology. 1995;108(4):1048-55. Epub 1995/04/01.

2. Ghoos YF, Maes BD, Geypens BJ, Mys G, Hiele MI, Rutgeerts PJ, et al. Measurement of gastric emptying rate of solids by means of a carbon-labeled octanoic acid breath test. Gastroenterology. 1993;104(6):1640-7. Epub 1993/06/01.

3. Veereman-Wauters G, Ghoos Y, van der Schoor S, Maes B, Hebbalkar N, Devlieger H, et al. The 13C-octanoic acid breath test: a noninvasive technique to assess gastric emptying in preterm infants. J Pediatr Gastroenterol Nutr. 1996;23(2):111-7.
